# The correlation of serum/plasma IGF-1 concentrations with obstructive sleep apnea hypopnea syndrome: A meta-analysis and meta-regression

**DOI:** 10.3389/fendo.2022.922229

**Published:** 2022-08-31

**Authors:** Jie He, Xiaoyan Li, Mi Yu

**Affiliations:** ^1^ Clinical Medical College of Chengdu Medical College, Chengdu, China; ^2^ Department of Pulmonary and Critical Care Medicine, The First Affiliated Hospital of Chengdu Medical College, Chengdu, China; ^3^ Department of Endocrinology, The First Affiliated Hospital of Chengdu Medical College, Chengdu, China

**Keywords:** obstructive sleep apnea hypopnea syndrome(OSAHS), meta-analysis, IGF-1, serum, plasma, correlation

## Abstract

**Background:**

Obstructive sleep apnea hypopnea syndrome (OSAHS) is a common disease that has serious cardiovascular and metabolic effects. Insulin-like growth factor 1 (IGF-1) levels are reportedly reduced in patients with OSAHS; however, this is still a matter of debate. Therefore, we investigated the association between serum/plasma IGF-1 levels and OSAHS in this meta-analysis.

**Methods:**

Wan Fang, Excerpta Medica dataBASE, Web of Science, China National Knowledge Infrastructure, VIP, PubMed, and other databases were searched for materials published in any language before April 2, 2022. Two researchers analyzed the studies for quality according to the Newcastle-Ottawa Scale. The acquired data were analyzed using Stata 11.0 and R 3.6.1 software. The effect size was estimated and calculated using standard mean differences and correlation coefficients. Moreover, a combined analysis was conducted using either a random- or fixed-effects model.

**Results:**

Ultimately, 34 studies met our inclusion criteria. Our findings revealed that the plasma/serum IGF-1 concentrations in patients with OSAHS was significantly reduced compared with those in healthy subjects. Subgroup analyses were performed according to OSAHS severity, ethnicity, age, body mass index, specimen testing method, and study design. The outcomes suggested that nearly all subgroups of patients with OSAHS had reduced serum IGF-1 levels. Disease severity and differences in ethnicity were identified as possible influencing factors of serum IGF-1 levels in patients with OSAHS in the meta-regression analysis, and no other factors were found to alter plasma/serum IGF-1 concentrations. Moreover, plasma/serum IGF-1 concentrations were negatively correlated with apnea-hypopnea index and oxygen desaturation index scores and positively associated with minimum oxygen saturation.

**Conclusion:**

Serum/plasma IGF-1 concentrations in patients with OSAHS were greatly reduced compared with those of patients in the control group, and were negatively correlated with apnea-hypopnea index and oxygen desaturation index scores and positively correlated with minimum oxygen saturation.

**Systematic Review Registration:**

https://www.crd.york.ac.uk/PROSPERO/, identifier CRD42022322738.

## 1 Introduction

The characteristic features of obstructive sleep apnea-hypopnea syndrome (OSAHS) include periodic, intermittent reductions in airflow (hypopnea) or no airflow (apnea) owing to complete or partial collapse of the upper respiratory tract during sleep (1). The respiratory physiological reactions to OSAHS include major alterations in intrathoracic pressure, intermittent hypoxemia, sleep fragmentation, and nocturnal awakening (2). Sleep patterns can change as a result of repeated awakenings, with reduced slow-wave sleep (SWS), rapid eye movement sleep, and enhanced light sleep (3). In the United States, 26% of people aged 30–70 years have had mild OSAHS, whereas 10% had moderate to severe OSAHS between 2007 and 2010 (4). OSAHS is more prevalent in men than women, and its incidence increases sharply with increasing body weight and age (4, 5).

OSAHS causes excessive daytime sleepiness, poor quality of life, poor job performance, and a higher risk of traffic accidents at a societal level (6). More importantly, OSAHS results in increased morbidity and mortality of comorbid conditions, such as coronary heart disease and cardiac failure, creating serious health risks for affected individuals (7). Hypertension, hyperlipidemia, insulin resistance, and metabolic syndrome are all risk factors for early cardiovascular disease, and the incidence of these diseases in patients with OSAHS is much higher than that in subjects matched by sex and body mass index (BMI) (8, 9).

In addition, OSAHS reportedly affects some hormonal systems, including catecholamines and the hypothalamic-pituitary axis. Sleep fragmentation related to awakening reportedly activates the sympathetic nervous system, which stimulates the release of nocturnal catecholamines (10, 11). Growth hormone (GH) secretion is closely associated with different sleep stages. GH is usually released in the form of pulses; two-thirds of GH secretion occurs during the first few hours of sleep, and the onset of pulses is linked to the stages of SWS (12). Accordingly, SWS disorder and shorter sleep times in patients with OSAHS may disrupt GH secretion at night. GH directly effects numerous tissues; however, many of its functions are achieved by increasing the levels of the second messenger, insulin-like growth factor-1 (IGF-1) (13). The liver produces most of the IGF-1 in the blood, which combines with IGF-associated binding proteins resulting in prolonged half-life and stable serum/plasma levels (14). The synergistic effect of GH and IGF-1 is important for the development of bones in children and helps maintain normal metabolism in adults (15).

According to some reports, serum/plasma IGF-1 concentration is negatively associated with apnea-hypopnea index (AHI) and respiratory awakening index scores among patients with OSAHS and positively associated with minimum oxygen saturation during sleep (16–18). Following reports of the above observation, the IGF-1 concentrations of patients with OSAHS became the focus of an increasing number of studies. Unfortunately, previous studies have shown conflicting results regarding decreased IGF-1 concentrations in patients with OSAHS. Furthermore, most studies included comparatively small sample sizes. Therefore, the current relevant research must be used for meta-analysis to evaluate the association between serum/plasma IGF-1 levels and the occurrence of OSAHS. To the best of our knowledge, the present analysis includes the most accessible literature. Furthermore, this meta-analysis and systematic review is the first to report the correlation coefficients (CORs) between OSAHS and AHI score, oxygen desaturation index (ODI) score, and minimum oxygen saturation, to better understand the potential function of serum/plasma IGF-1 in patients with OSAHS.

## 2 Methods

### 2.1 Determination of qualified literature and data screening

Wan Fang, Excerpta Medica dataBASE, China National Knowledge Infrastructure, VIP, Web of Science, and PubMed databases were systematically searched to find relevant studies published until April 2, 2022. The keywords and subject terms used included “Insulin-Like Growth Factor 1” or “IGF-1” and “Obstructive Sleep Apnea-Hypopnea Syndrome” or “Obstructive Sleep Apnea” or “Obstructive Sleep Apnea Syndrome” or “OSA” or “OSAHS” or “OSAS.” The eligibility criteria were as follows:

1. Case-control, cohort, or cross-sectional study.
2. Serum/plasma levels of IGF-1 were analyzed in patients with OSAHS and healthy people of all sexes, nationalities, ages, and ethnicities.
3. Subjects met the diagnostic criteria for OSAHS based on polysomnography (PSG) (adults: AHI ≥5/h; children: AHI ≥1/h) (19, 20),

OSAHS severity was defined using traditional definitions (AHI<5, normal; AHI 5–14, mild OSAHS; AHI 15–29, moderate OSAHS; and AHI ≥30, severe OSAHS) (21). The exclusion criteria were as follows:

1. Editorials, reviews, letters, other types of literature reviews, or case report.
2. Unable to extract enough information from the original article or contact the corresponding author for more information.
3. Studies that were not conducted in humans.
4. Studies including patients with OSAHS with a history of chronic airway disease, cerebrovascular disease, chronic cardiac failure, endocrine disease, and malignancies.
5. Studies lacking a control group.
6. Studies reporting controls with AHI ≥5 events/h in adults and AHI ≥1 events/h in children.
7. Overlapping studies and overlapping data from studies by the same authors.

### 2.2 Literature selection

Based on the aforementioned data retrieval methods, two authors separately searched the databases, and the titles and abstracts of relevant articles were reviewed. We made a preliminary list of all eligible full-text papers. Subsequently, we re-evaluated the publications that met the inclusion criteria by carefully reading and reviewing the full text. Upon disagreement between the two authors regarding article eligibility, a third expert researcher was consulted to resolve the dispute by consensus.

### 2.3 Data extraction and management

We created some special tables to extract the following information from each eligible study as follows:

1. Basic data, such as the publication date and first author’s name.
2. Baseline features of study participants, such as BMI, age, sample size, sex, ethnicity, study design, classification of sleep breath disorder, and serum/plasma IGF-1 concentrations in patients as well as controls. We converted the data into means and standard deviations using an online computing tool if the selected studies provided data on the median and range or median and interquartile range (22, 23) (https://www.math.hkbu.edu.hk/~tongt/papers/median2mean.html).
3. Disease severity.
4. Literature quality scores
5. Spearman’s rank COR, or Pearson’s COR for AHI score, ODI score, minimum oxygen saturation, and IGF-1 concentration.

### 2.4 Methodological evaluation of research quality

The Newcastle-Ottawa Scale (24) was used to assess the quality of the selected papers as follows: study population (four items, total score 4), exposure or outcome (three items, total score 3), and comparability (one item, total score 2). Total scores of 7–9, 4–6, and 0–3 were considered high-, medium-, and low-quality studies, respectively.

### 2.5 Statistical analysis

R (version 3.6.1; R Foundation for Statistical Computing, Vienna, Austria) and Stata software (version 11.0; StataCorp LLC, College Station, TX, USA) were used to summarize and examine the extracted data. We normalized and expressed the continuous variables as the standardized mean difference (SMD) with a 95% confidence interval (95%CI). The current meta-analysis used Spearman’s CORs to examine the associations between IGF-1 concentrations and PSG indices in patients with OSAHS. According to the standard error, which is mostly dependent on the importance of the rank COR, the dependence of the Spearman’s product-moment COR on the sampling distribution is not indicated. Therefore, the Fisher transformation was used to compare each COR, and an investigation was subsequently conducted with the transformed values as the input before converting them back to CORs. Cohen’s criteria was used to examine the measured effect size (small, ≤0.3; moderate, 0.3–0.5; and large, >0.5 (25). Pearson’s COR was used to examine the relationships among AHI score, ODI score, minimum oxygen saturation, and IGF-1 levels.

In accordance with the above description, several studies have reported a method for converting Pearson’s to Spearman’s COR using the following formula:


$$r=2\text{sin}(r_{s}\frac{\pi }{6})$$

where r and r_s_ represent the Pearson’s and Spearman’s CORs, respectively (26). Cochran’s Q and chi-square tests were used to examine data heterogeneity. The I^2^ statistic was used to detect heterogeneity (25%, 50%, and 75% represented low, moderate, and high heterogeneity, respectively; I^2 ^< 50% and I^2 ^> 50% indicated low and high heterogeneity among studies, respectively). In the case of zero heterogeneity among studies, we employed fixed- and random-effects models if heterogeneity was not noted among studies.

Descriptive analysis, subgroup analysis, and meta-regression were used to explore the source of heterogeneity. For subgroup analyses, the overall population was categorized according to disease severity, ethnicity, method of specimen testing, study design, age, and BMI. One study at a time was removed for the sensitivity analysis, which was conducted to explore how each study impacted the combined effect size. Begg’s and Egger’s tests were performed to assess for publication bias.

## 3 Results

### 3.1 Publications retrieved and included in the study

**Figure 1:**
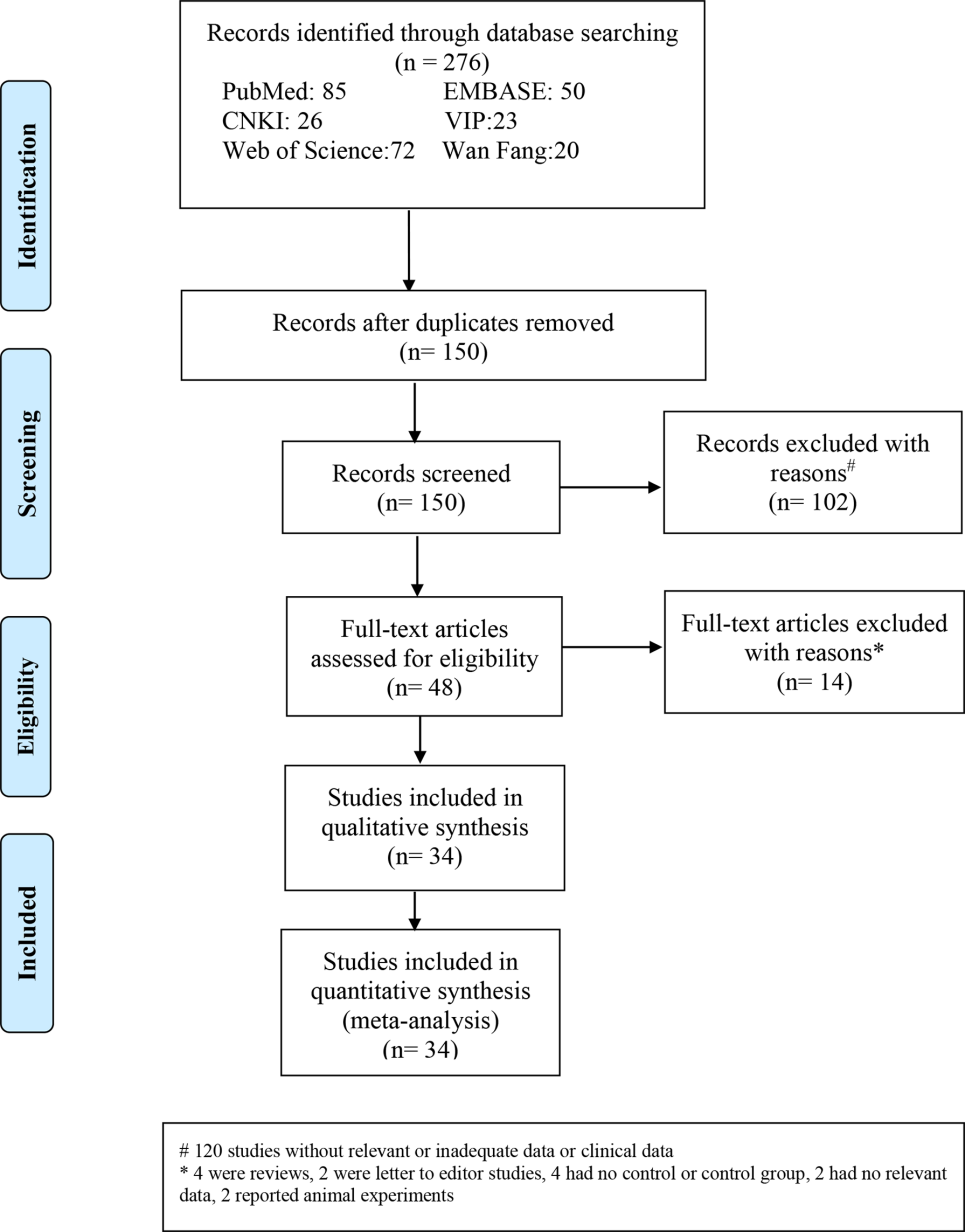
Flow diagram indicating the literature selection process and results based on the preferred reporting items for the meta-analysis.

A total of 276 related papers were collected from the databases. Duplicated studies were excluded by filtering the abstracts and titles; 228 articles were omitted from the study, leaving 48 articles. We downloaded these 48 articles and thoroughly reviewed the complete text; 14 papers were discarded following review of the inclusion and exclusion criteria. Articles were excluded for the following reasons: four publications were reviews, two were letters to the editor, four did not include a control group of healthy people, two lacked relevant data, and two were animal experiments. We identified 34 studies (34 studies from 21 articles) (17, 18, 27–45) involving IGF-1 levels in the plasma/serum, with 8 publications reporting plasma IGF-1 concentrations and 26 publications reporting serum IGF-1 concentrations. We selected 2 cohort studies, 10 cross-sectional studies, and 22 case-control studies were selected, as shown in **Table 1**. **Table 2** summarizes the data on age, IGF-1 concentrations, BMI, severity, and AHI scores. Twelve studies provided Spearman’s or Pearson’s CORs between IGF-1 concentration and AHI score, ODI score, and minimum oxygen saturation. **Figure 1** shows the Preferred Reporting Items for Systematic Reviews and Meta-Analyses flow diagram for selecting and screening articles from the literature. **Tables 1**– **3** present the fundamental data of the included studies.

**Table 1:** Characteristics of included studies.

Author	Year	Country	Ethnicity	Case/Control (n)	IGF-1 Source	Assay approach	NOS	Study design	Measurement and type
Damanti S	2017	France	Caucasian	47/25	Plasma	Chemiluminescence	7	Cross-sectional study	PSG II
Makino S (Mild and Moderate)	2012	Japan	Asian	88/34	Plasma	ELISA	7	Cross-sectional study	PSG I
Makino S(Severe)	2012	Japan	Asian	69/34	Plasma	ELISA	7	Cross-sectional study	PSG I
Gozal D	2008	USA	Caucasian	87/23	Plasma	ELISA	7	Case-control study	PSG I
Barceló A	2008	Spain	Caucasian	22/23	Plasma	Chemiluminescence	7	Case-control study	PSG I
Ursavas A	2007	Turkey	Caucasian	39/36	Plasma	Chemiluminescence	7	Cross-sectional study	PSG I
McArdle N	2007	Australia	Caucasian	21/21	Plasma	ELISA	7	Case-control study	PSG I
Nieminen P	2002	Finland	Caucasian	30/35	Plasma	Radioimmunoassay	7	Case-control study	PSGIII
Martínez Cuevas E	2021	Spain	Caucasian	36/31	Serum	ELISA	7	Cohort study	PSG I
Zhao X (Mild and Moderate)	2021	China	Asian	18/13	Serum	Chemiluminescence	7	Cross-sectional study	PSG I
Zhao X (Severe)	2021	China	Asian	18/13	Serum	Chemiluminescence	7	Cross-sectional study	PSG I
Wang X	2018	China	Asian	192/100	Serum	Chemiluminescence	7	Cross-sectional study	PSG I
Guo X	2018	China	Asian	13/12	Serum	Chemiluminescence	7	Cross-sectional study	PSG I
Kanbay A	2017	Turkey	Caucasian	33/17	Serum	Radioimmunoassay	7	Cross-sectional study	PSG I
Izumi S (Mild)	2016	Brazil	Latino	8/11	Serum	Chemiluminescence	7	Cross-sectional study	PSG I
Izumi S (Moderate and Severe)	2016	Brazil	Latino	28/11	Serum	Chemiluminescence	7	Cross-sectional study	PSG I
Gianotti L	2002	Italy	Caucasian	13/15	Serum	Radioimmunoassay	7	Cohort study	PSG I
Xie CB (Mild)	2018	China	Asian	31/50	Serum	ELISA	7	Case-control study	PSG I
Xie CB (Moderate)	2018	China	Asian	32/50	Serum	ELISA	7	Case-control study	PSG I
Xie CB (Severe)	2018	China	Asian	44/50	Serum	ELISA	7	Case-control study	PSG I
Chen XL (Mild)	2020	China	Asian	42/50	Serum	ELISA	7	Case-control study	PSG I
Chen XL (Moderate)	2020	China	Asian	56/50	Serum	ELISA	7	Case-control study	PSG I
Chen XL (Severe)	2020	China	Asian	20/50	Serum	ELISA	7	Case-control study	PSG I
Qu BB	2016	China	Asian	80/80	Serum	ELISA	7	Case-control study	PSG I
Zhou JJ (Mild)	2018	China	Asian	82/40	Serum	Chemiluminescence	7	Case-control study	PSG I
Zhou JJ (Moderate)	2018	China	Asian	78/40	Serum	Chemiluminescence	7	Case-control study	PSG I
Zhou JJ (Severe)	2018	China	Asian	60/40	Serum	Chemiluminescence	7	Case-control study	PSG I
Zhang W (Mild)	2019	China	Asian	15/15	Serum	Chemiluminescence	7	Case-control study	PSG I
Zhang W (Moderate)	2019	China	Asian	15/15	Serum	Chemiluminescence	7	Case-control study	PSG I
Zhang W (Severe)	2019	China	Asian	15/15	Serum	Chemiluminescence	7	Case-control study	PSG I
Lou F (Mild)	2015	China	Asian	14/10	Serum	Chemiluminescence	7	Case-control study	PSG I
Lou F (Moderate)	2015	China	Asian	19/10	Serum	Chemiluminescence	7	Case-control study	PSG I
Lou F (Severe)	2015	China	Asian	17/10	Serum	Chemiluminescence	7	Case-control study	PSG I
Hashim Z	2022	india	Caucasian	25/10	Serum	Chemiluminescence	7	Case-control study	PSG I

NOS, Newcastle-Ottawa scale; ELISA, Enzyme linked immunosorbent assay.

**Table 2:** Participants’ characteristics of included studies.

Author	IGF-1 (Mean±SD)		BMI (Mean±SD)		Age (Mean±SD)		AHI (Mean±SD)	
	Case	Control	Case	Control	Case	Control	Case	Control
Damanti S	101.42±67.32	164.99±93.88	26.8±4.4	24.3±4.5	75.2±8.1	73.1±7.1	35.1±15.7	8.3±4.3
Makino S (Mild and Moderate)	176.2±5.3	194±8.1	25.6±0.7	24.8±1.1	49.8±12.7	46.32±12.77	24.6±2.4	2.2±0.8
Makino S(Severe)	165±5.3	194±8.1	28.5±1.1	24.8±1.1	47.1±11.3	46.32±12.77	77.1±10.3	2.2±0.8
Gozal D	1070±240	840±70	18.3±0.5	17.4±0.6	6.4±0.4	6.2±0.5	10.0±2.0	0.8±0.2
Barceló A	106±27	122±36	31±4	25±3	50±5	48±6	52±19	3±1
Ursavas A	89.8±39.2	140.1±54.5	33.6±7.2	30.5±6.5	52.0±9.6	48.8±10.3	50.5±23.5	1.9±1.2
McArdle N	123.59±39.75	143.59±39.63	28.4±3.4	27.9±3.6	46±10.2	46±9.7	40±27	2.8±1.5
Nieminen P	11.01±1.89	11.11±2.0	15.93±1.17	16±0.77	5.63±1.07	6.45±1.27	6.14±2.52	NA
Martínez Cuevas E	101.42±67.32	164.99±93.88	NA	NA	4.97±2.38	6.41±2.64	NA	NA
Zhao X (Mild and Moderate)	421.3±113.7	477.9±161	25.5±2.8	25.7±3.3	40.2±10.1	34.1±10.5	16.6±7.4	2.3±1.8
Zhao X (Severe)	438.9±124.2	477.9±161	27.6±4.0	25.7±3.3	48.1±9.7	34.1±10.5	62±20	2.3±1.8
Wang X	64.74±22.89	78.39±34	28.37±3.89	24.1±3.27	44.15±11.49	46.24±12.23	49.3±21.16	6.32±4.83
Guo X	954.2±203	1045±317.6	27.1 ± 3.1	25.5 ± 3.7	47.0 ± 8.5	35.1 ± 9.5	23.77±44.35	NA
Kanbay A	79.1±36.1	147.1±49.1	35.4±5.7	31.5±4.3	51±9	47±6	46.97±25.96	1.48±0.89
Izumi S (Mild)	177.2±41.4	225.5±80.5	34.6±6.2	31.9±2.9	51±9	42.3±8.3	8.93±6.7	2±2.29
Izumi S (Moderate and Severe)	156.8±54.3	225.5±80.5	33±3.2	31.9±2.9	45.0±3.03	42.3±8.3	39.06±26.4	2±2.29
Gianotti L	17.5±1.9	21.3±1.6	38.5±2.9	22.2±0.6	52.6±2.8	39.6±0.9	51.6±9.2	2.2±0.6
Xie CB (Mild)	65.48±4.88	68.73±5.47	26.35±3.66	24.76±4.01	46.1±7.7	45.9±8.2	10.77±5.08	3.57±2.41
Xie CB (Moderate)	52.77±6.21	68.73±5.47	25.29±3.21	24.76±4.01	47.2±6.5	45.9±8.2	24.93±4.77	3.57±2.41
Xie CB (Severe)	43.84±5.96	68.73±5.47	26.03±4.13	24.76±4.01	48.7±9.6	45.9±8.2	48.77±10.51	3.57±2.41
Chen XL (Mild)	471.28±73.81	549.37±71.09	NA	NA	45.37±12.95	45.05±11.72	NA	NA
Chen XL (Moderate)	350.26±65.77	549.37±71.09	NA	NA	44.81±11.75	45.05±11.72	NA	NA
Chen XL (Severe)	198.04±35.83	549.37±71.09	NA	NA	46.11±13.06	45.05±11.72	NA	NA
Qu BB	77.33±22.15	50.25±19.95	26.21±1,65	26.08±1.33	50.21±11.22	53.12±10.05	NA	NA
Zhou JJ (Mild)	147.63±26.83	149.44±31.44	15.6±1.27	15.84±2.35	4.5±0.84	4.47±0.89	NA	NA
Zhou JJ (Moderate)	142.11±34.5	149.44±31.44	15.38±0.96	15.84±2.35	4.49±0.84	4.47±0.89	NA	NA
Zhou JJ (Severe)	121.86±36.47	149.44±31.44	15.52±1.37	15.84±2.35	4.49±0.87	4.47±0.89	NA	NA
Zhang W (Mild)	70.25±8.55	75.03±10.1	24.38±1.56	21.18±1.36	52.6±6.9	53.1±6.6	11.55±2.25	3.75±1.12
Zhang W (Moderate)	54.28±8.25	75.03±10.1	27.58±1.72	21.18±1.36	54.8±5.8	53.1±6.6	20.68±4.65	3.75±1.12
Zhang W (Severe)	42.25±6.95	75.03±10.1	30.78±2.02	21.18±1.36	55.1±7.7	53.1±6.6	45.95±8.09	3.75±1.12
Lou F (Mild)	166.43±44.46	192.1±61.51	NA	NA	NA	NA	NA	NA
Lou F (Moderate)	160.06±57.14	192.1±61.51	NA	NA	NA	NA	NA	NA
Lou F (Severe)	114.60±33.24	192.1±61.51	NA	NA	NA	NA	NA	NA
Hashim Z	516.6±415.97	571.1±283.4	28.3±5.5	26.3±6.6	41.4±13.4	33.6±12.4	16.91±18	7.86±7.84

NA, not available.

**Table 3:** Correlation coefficients (cor‐values) of included studies.

Author	Year	N	Cor of IFG-1 versus AHI	Cor of IFG-1 versus ODI	Cor of IFG-1 versus Minimum oxygen saturation
Makino S	2012	157	-0.226		0.259
Gozal D	2008	87	0.12		0.19
Izumi S	2016	36	-0.312		0.323
Ursavas A	2007	39	-0.42	-0.224	
Wang X	2018	192	-0.141	-0.116	
Xie CB	2018	107	-0.641		0.506
Chen XL	2020	118	-0.633		
Zhang W	2019	45	-0.296		
Lou F(Mild)	2015	14	-0.334		0.207
Lou F(Moderate)	2015	19	-0.02		0.61
Lou F(Severe)	2015	17	-0.505		0.553
Kanbay A	2017	33		-0.486	0.452

AHI, Apnea-hypopnea index.

ODI, Oxygen-desaturation index.

### 3.2 Literature quality assessment

We used Newcastle-Ottawa Scale scores to help assess the methodological quality of the studies selected for this analysis. Every article in this study had a score > 6, suggesting that the quality of the included publications was comparatively high. We included 30 high-quality and 4 medium-quality publications.

### 3.3 Meta-analysis

#### 3.3.1 IGF-1 concentrations in all patients with OSAHS

**Figure 2:**
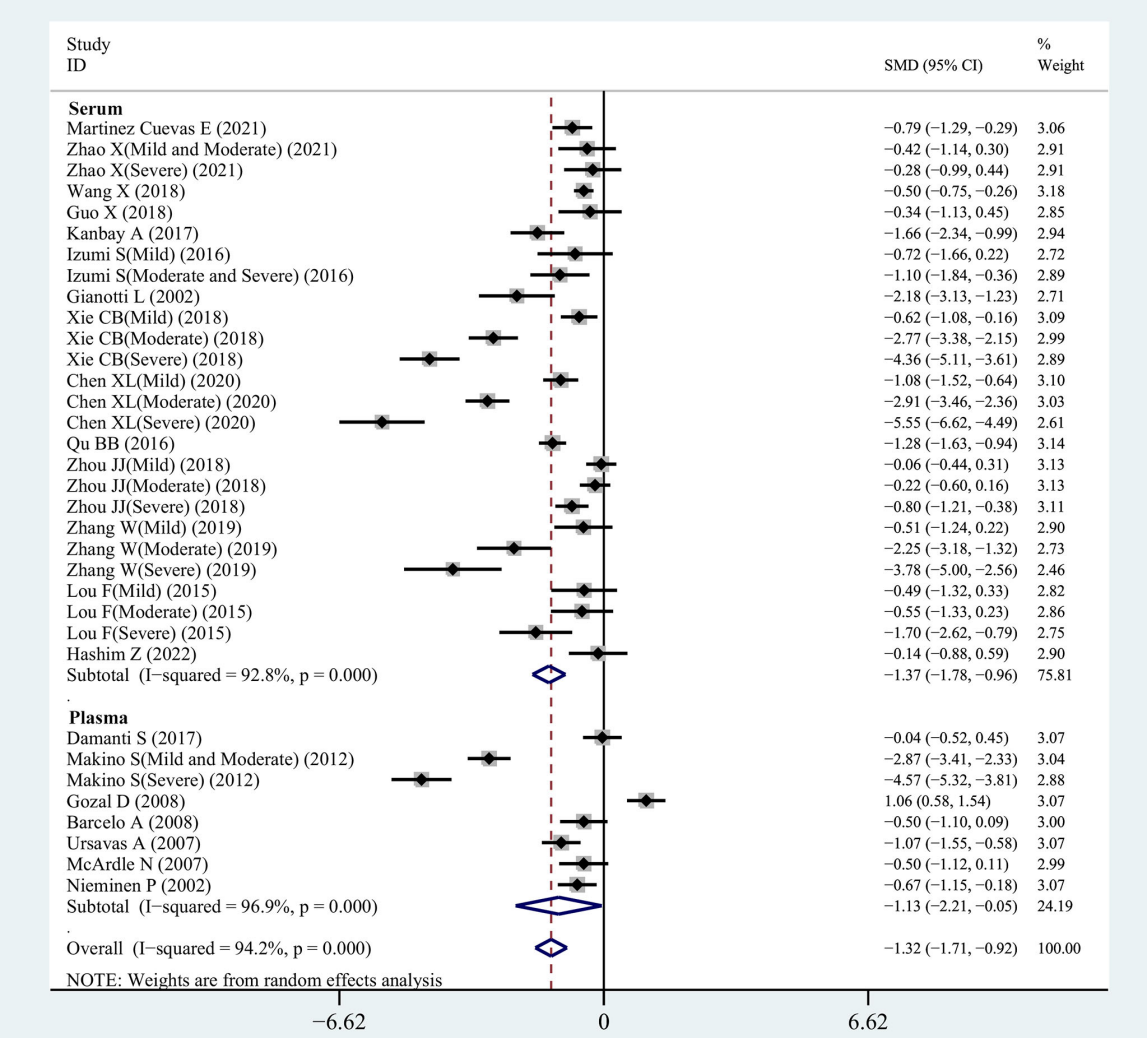
SMD forest plot and its 95%CI for serum/plasma IGF-1 measures in the OSAHS group and the control group.

**Figure 3:**
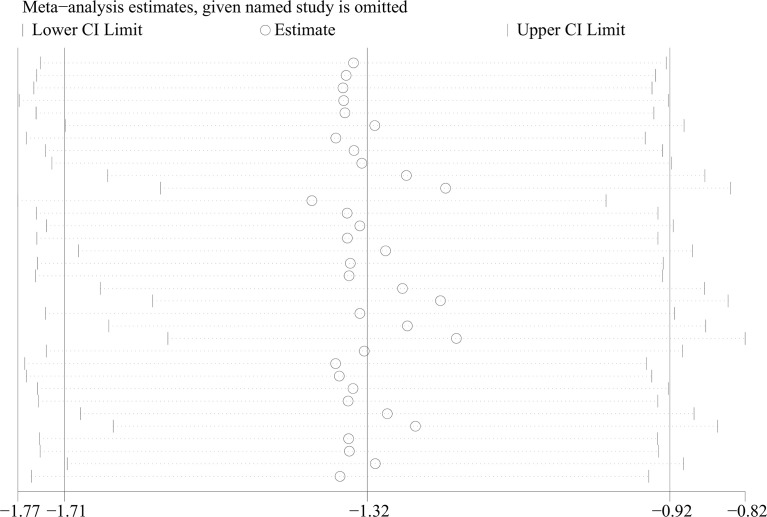
Sensitivity analysis of studies on IGF-1 levels for OSAHS versus controls.

From 34 studies, we identified 1407 patients with OSAHS and 1039 healthy controls. The heterogeneity index I^2^ was 94.2%. Therefore, we opted for a random-effects model for data combination. The meta-analysis outcomes revealed that IGF-1 levels in patients with OSAHS were significantly lower than those of healthy controls (SMD= -1.32, 95%CI= -1.71–0.92, P< 0.001; **Figure 2**). Moreover, we performed a series of sensitivity assessments to determine the reliability of the merged data. Major alterations were not detected in the meta-analysis outcomes after omitting the included studies individually, thereby proving their reliability (**Figure 3**). Because different sample types may cause heterogeneity in the analysis outcomes, we assessed the changes in serum/plasma IGF-1 concentrations in patients with OSAHS.

#### 3.3.2 Comparison of Serum IGF-1 concentrations among patients in OSAHS and control groups

The correlation between serum IGF-1 concentrations in patients with OSAHS and those of patients in the control group was investigated in our meta-analysis, which included 26 eligible observational studies. Serum IGF-1 levels were greatly reduced in patients with OSAHS (SMD=-1.37, 95%CI= -1.78–0.96, P< 0.001). For further investigation, we selected a random-effects model owing to increased heterogeneity (I^2 =^ 92.8%, **Figure 2**).

#### 3.3.3 Comparison of plasma IGF-1 concentrations between OSAHS and control groups

The outcomes of the pooled analysis of plasma IGF-1 concentrations in patients with OSAHS are shown in **Figure 2**. The results indicated that plasma IGF-1 concentrations in patients with OSAHS were significantly lower than those of patients in the control group (SMD=-1.13, 95%CI= -2.21–0.05, P=0.04). We selected a random-effects model for further investigation owing to high heterogeneity (I^2 =^ 96.9%).

### 3.4 Subgroup analysis of plasma IGF-1 concentrations

#### 3.4.1 Age

Plasma IGF-1 levels in children with and without OSAHS were studied in four investigations. According to the findings, there was no major variation in plasma IGF-1 levels between children with OSAHS and those of children in the control group (SMD=0.20, 95%CI= -1.71–1.89, P=0.818). Four studies compared plasma IGF-1 concentrations in adults with and without OSAHS, with lower plasma IGF-1 levels reported in adults with OSAHS (SMD=-1.58, 95%CI= -2.82–0.33, P< 0.001, **Table 4**).

**Table 4:** Subgroup analyses of IGF-1 levels in OSAHS and controls.

Subgroup analysis of plasma levels (n)	SMD(95% CI), *P*-value, I^2^ (%), *P*_h_	Subgroup analysis of serum levels (n)	SMD(95% CI), *P*-value, I^2^ (%), *P*_h_
Overall (8)		Overall (26)	
Ethnicity		Ethnicity	
Caucasian (6)	-0.20 (-0.91,0.35), 0.383, 88.70% , <0.0001	Caucasian (4)	-1.15 (-1.94,-0.37), 0.004, 80.70%, 0.001
Asian (2)	-3.70 (-5.36,-2.04), <0.0001, 92.20% , <0.0001	Asian (20)	-1.46 (-1.96,-0.97), <0.001, 94.20%, <0.0001
Latino		Latino (2)	-0.95 (-1.54,-0.37), 0.001, 0.0%, 0.533
Assay approaches		Assay approaches	
ELISA (4)	-1.71 (-4.11,0.69), 0.163, 98.50%, <0.0001	ELISA (8)	-2.36 (-3.39,-1.43), <0.0001, 96.00%, <0.0001
Chemiluminescence (3)	-0.54 (-1.16,0.09) ,0.093, 77.00%, 0.013	Chemiluminescence (16)	-0.74 (-1.04,-0.43), <0.0001, 75.40%, <0.0001
Radioimmunoassay (1)	NA	Radioimmunoassay (2)	-1.84 (-2.38,-1.29), <0.0001, 0.0%, 0.386
BMI		BMI	
Mean BMI≥30 (2)	-0.81 (-1.36,-0.26), 0.004 , 52.10%, <0.0001	Mean BMI≥30 (5)	-1.81 (-2.66,-0.97), <0.0001, 93.60%, <0.0001
Mean BMI<30 (6)	-0.135 (-2.73,0.23), 0.10, 97.70%, 0.148	Mean BMI<30 (21)	-1.27 (-1.73,-0.82), <0.0001, 78.70%, 0.001
Degree of severity		Degree of severity	
Mean AHI≥30 (5)	-1.32 (-2.64,0.005), 0.05, 96.30%, <0.0001	Mean AHI≥30 (10)	-2.14 (-3.05,-1.22), <0.0001, 95.40% , <0.0001
Mean AHI<30 (3)	-0.82 (-3.01,1.37), 0.46, 98.30%, <0.0001	Mean AHI<30 (16)	-0.95 (-1.38,-0.51), <0.0001, 89.60% , <0.0001
Age		Age	
Adult (6)	-1.58 (-2.82,-0.33), 0.013, 96.60%, <0.0001	Adult (19)	-1.66 (-2.21,-1.12), <0.0001, 93.80%, 0.0001
Nonage (2)	0.20 (-1.49,1.89), 0.818 , 95.90%, <0.0001	Nonage (7)	-0.58 (-0.92,-0.23), 0.001, 64.90%, 0.009
Design		Design	
Cross-sectional study (4)	-2.12 (-3.89,-0.34), 0.019, 97.60%, <0.0001	Cross-sectional study (6)	-0.52 (-0.72,-0.32), <0.0001, 0.0%, 0.662
Case-control study (4)	-0.14 (-1.01,0.72), 0.745, 90.30%, <0.0001	Case-control study (18)	-1.65 (-2.23,-1.08), <0.0001, 94.50%, <0.0001
Cohort study	NA	Cohort study (2)	-1.42 (-2.78,-0.07), 0.040, 84.5%, 0.816

NA, not available.

#### 3.4.2 BMI

Subgroup analysis was performed according to mean BMI (> 30), which was reported in all included studies. Two studies reported plasma IGF-1 concentrations in patients with a mean BMI > 30, suggesting that plasma IGF-1 concentrations in patients in the OSAHS group were lower than those of patients in the healthy control group (SMD=-0.81, 95%CI= -1.36–0.26, P=0.004). According to six studies, plasma IGF-1 concentrations in patients with a mean BMI< 30 showed no major variations between patients in the OSAHS and control groups (SMD=-0.135, 95%CI= -2.73–0.23, P=0.46, **Table 4**).

#### 3.4.3 OSAHS severity

A subgroup meta-analysis of OSAHS severity was also performed. Plasma IGF-1 levels were measured in five patients with an mean AHI score ≥30 and three studies reported plasma IGF-1 levels in patients with an mean AHI score< 30. The outcomes revealed that regardless of mean AHI score, there were no major variations in plasma IGF-1 levels between patients in the OSAHS and control groups (SMD=-1.32, 95%CI= -2.64–0.005, P=0.05; SMD=-0.82, 95%CI=-3.01–1.37, P=0.46, **Table 4**).

#### 3.4.4 Assay approaches

A subgroup analysis was performed according to the different detection techniques used in the samples because variations in detection techniques for plasma IGF-1 levels may cause errors in the outcome analysis. In four studies, plasma IGF-1 levels were calculated using an enzyme-linked immunosorbent assay. The findings indicated no major variations in plasma IGF-1 concentrations between the OSAHS and control groups (SMD=-1.32, 95% CI = -2.64–0.005, P=0.05). In three studies, plasma IGF-1 concentrations were calculated using chemiluminescence, which also revealed no major differences in plasma IGF-1 concentrations between OSAHS and control groups (SMD=-0.54, 95% CI = -1.16–0.09, P=0.093). **Table 4** summarizes these findings. One study used radioimmunoassay to determine plasma IGF-1 concentrations and reported the same results as with the above assays; there were no major differences in plasma IGF-1 concentrations between 30 patients with OSAHS and 35 healthy subjects (P=0.92).

#### 3.4.5 Ethnicity

The results of the subgroup analysis of plasma IGF-1 concentrations in patients with OSAHS of different ethnicities are compiled in **Table 4**. The Caucasian and Asian populations were the major subgroups. No major variations in plasma IGF-1 concentrations were observed between the OSAHS and control groups (SMD=-0.20, 95%CI=-0.91–0.35, P=0.383) in the Caucasian population. In the Asian subgroup, serum IGF-1 levels were lower in the OSAHS group than those in the control group (SMD=-3.70, 95%CI= -5.36–2.04, P< 0.001, **Table 4**).

#### 3.4.6 Research design

We conducted a matched subgroup analysis because of the differences in study designs of the included studies, which might alter the heterogeneity of the results. Plasma IGF-1 concentrations in patients with OSAHS were reduced compared with those of subjects in the control group (SMD=-2.12, 95 percent CI= -3.89–0.34, P=0.019), according to a subgroup analysis of cross-sectional studies. No major variations were observed in plasma IGF-1 concentrations between patients in the OSAHS and control groups (SMD=-0.14, 95%CI= -1.01–0.72, P=0.745) according to a subgroup analysis of case-control studies (**Table 4**).

### 3.5 Subgroup analysis of serum IGF-1 concentrations

#### 3.5.1 Age

The serum IGF-1 levels of children with OSAHS and healthy children were evaluated in seven investigations. The combined results revealed that serum IGF-1 levels in children with OSAHS were considerably lower than those in healthy children (SMD=-0.58, 95% CI=-0.92–0.23, P=0.001). The blood IGF-1 concentrations of adults with OSAHS were studied in 19 investigations, and the combined results revealed that serum IGF-1 concentrations in adults with OSAHS were considerably lower than those of participants without OSAHS (SMD=-1.66, 95%CI= -2.21–1.12, P 0.001, **Table 4**).

#### 3.5.2 BMI

We conducted a subgroup meta-analysis according to BMI in 26 studies. Five studies were based on serum IGF-1 concentrations in patients with mean BMI≥30, and the analysis revealed that these patients had greatly reduced IGF-1 concentrations compared with those of patients in the control group (SMD=-1.81, 95%CI=-2.66–0.97, P< 0.001). Twenty-one publications provided data on serum IGF-1 concentrations in patients with a mean BMI< 30, and the analysis indicated that these patients had significantly lower IGF-1 concentrations than those of patients in the control group (SMD=-1.27, 95%CI= -1.73–0.82, P< 0.001, **Table 4**).

#### 3.5.3 OSAHS severity

Many studies reported a close association between serum IGF-1 concentrations and AHI scores in patients with OSAHS. In the current study, we compared serum IGF-1 concentrations between patients with OSAHS with an mean AHI score ≥30 and those of patients in the control group in 10 studies. The findings suggested that serum IGF-1 levels in these patients were greatly reduced compared with those of patients in the control group (SMD=-2.14, 95%CI= -1.73–0.82, P< 0.001). Sixteen studies were based on comparisons of serum IGF-1 concentrations between patients with OSAHS with a mean AHI score< 30 and those of patients in the control group, which indicated that serum IGF-1 concentrations in these patients were greatly reduced compared with those of subjects in the control group (SMD=-0.95, 95%CI= -1.38–0.51, P< 0.001, **Table 4**).

#### 3.5.4 Assay approaches

The enzyme-linked immunosorbent assay results of eight reports suggested that serum IGF-1 levels were decreased in patients with OSAHS compared with those of patients in the control group (SMD=-2.36, 95%CI= -3.39–1.43, P< 0.001). We checked the serum IGF-1 concentration in 16 studies using chemiluminescence and found that serum IGF-1 concentrations in patients with OSAHS were greatly reduced compared with those of subjects in the control group (SMD=-0.74, 95%CI= -1.04–0.43, P< 0.001). In two studies, serum IGF-1 concentrations were studied using radioimmunoassay, and similar results were obtained (SMD=-1.84, 95%CI= -2.38–1.29, P< 0.001, **Table 4**).

#### 3.5.5 Ethnicity

We included 26 reports on Caucasian, Asian, and Latin American populations in this study. The serum IGF-1 concentrations of patients with OSAHS among these three groups were all lower than those of patients in the respective control groups in subgroup analyses based on varying demographics and ethnicities (see **Table 4**).

#### 3.5.6 Research design type

Among cross-sectional studies, blood IGF-1 concentrations in patients with OSAHS were substantially higher than those of subjects in the control group (SMD=-0.52, 95%CI=-0.72–0.32, P 0.001). Similarly, serum IGF-1 concentrations were lower in patients with OSAHS than those in patients in the control group in both case-control and cohort studies (SMD=-1.65, 95%CI= -2.23–1.08, P 0.001; SMD=-1.42, 95%CI=-2.78–0.07, P 0.001, **Table 4**).

### 3.6 Meta-analysis of correlation between plasma/serum IGF-1 concentration and ODI score, AHI score, and minimum oxygen saturation

**Figure 4:**
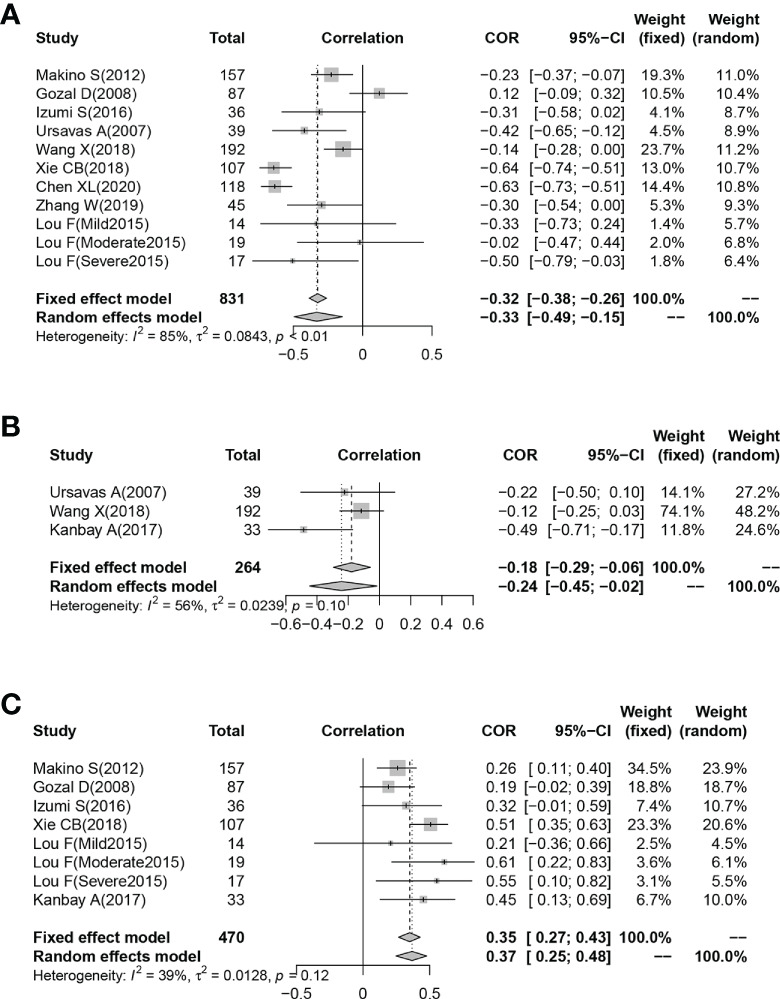
Funnel plot of effect sizes measured as correlations between serum/plasma IGF-1 levels and AHI, ODI and minimum oxygen saturation. **(A)** AHI; **(B)** ODI; **(C)** minimum oxygen saturation.

Eleven studies reported Pearson’s or Spearman’s CORs for the correlation between plasma/serum IGF-1 concentrations and AHI scores. Three studies reported Pearson’s or Spearman’s CORs for the correlation between ODI score and serum/plasma IGF-1 levels in patients with OSAHS. Eight studies reported Pearson’s or Spearman’s CORs for the relationship between minimum oxygen saturation and serum/plasma IGF-1 levels in patients with OSAHS. The ODI score, AHI score, and minimum oxygen saturation are important criteria for evaluating OSAHS, and AHI scores directly correlate with OSAHS severity. We conducted a meta-analysis on serum/plasma IGF-1 concentrations, as well as AHI score, ODI score, and minimum oxygen saturation in patients with OSAHS using the “meta” R package. The analysis revealed an effect size for serum/plasma IGF-1 concentration and AHI score of -0.33 (95%CI= -0.49–0.15, P< 0.001) (**Figure 4A**). The effect size for serum/plasma IGF-1 concentration and ODI score was -0.24 (95%CI= -0.49–0.15, P< 0.001). P< 0.001) (**Figure 4B**) and 0.37 (95%CI= 0.25–0.48, P< 0.001) for serum/plasma IGF-1 concentration and minimum oxygen saturation (**Figure 4C**).

### 3.7 Meta-regression

All included studies had an I^2^ value of 94.2%, indicating a high level of heterogeneity. Therefore, we explored the possible sources of heterogeneity using meta-regression. **Table 5** shows the meta-regression of serum/plasma IGF-1 concentrations. Meta-regression suggested that ethnicity may influence plasma IGF-1 concentration (R=-3.396, P=0.003). In the meta-regression of serum IGF-1 concentrations, disease severity was also a possible factor affecting serum IGF-1 levels (R=-1.288, P=0.027). Serum IGF-1 levels were more reduced in patients with severe OSAHS compared with patients with mild or moderate OSAHS. This heterogeneity was not caused by the sample detection method, age, or study design.

**Table 5:** Meta-regression analysis of variables predicting serum and plasma levels of IGF-1.

Variables	R	Adjusted R^2^	P
Age			
Serum	- 1.025	0.086	0.095
Plasma	- 1.778	0.094	0.241
BMI			
Serum	-0.561	-0 017	0 439
Plasma	0.465	-0. 157	0.773
Severity			
Serum	- 1.288	0. 163	0.027
Plasma	- 0.499	-0. 149	0.728
Ethnicity			
Serum	0.013	-0.048	0.982
Plasma	-3.396	0.793	0.003
Assay approaches			
Serum	-2.820	-0.001	0.220
Plasma	0.706	-0.068	0.472
Design			
Serum	-0.695	0.037	0.182
Plasma	1.963	0.257	0.118

### 3.8 Publication bias

**Figure 5:**
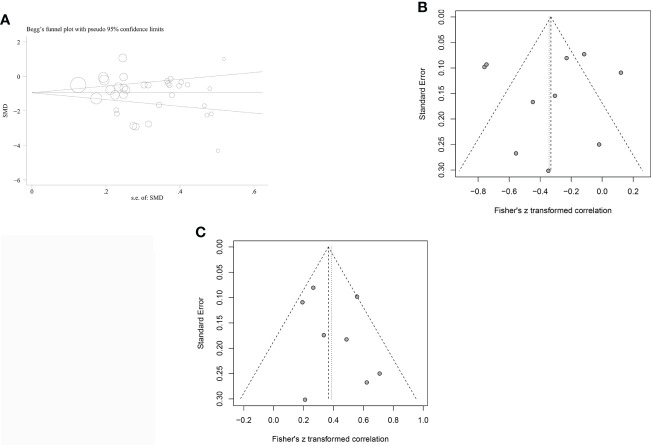
Funnel plots were employed to assess the publication bias among the included studies examining the relationship of leptin levels with OSAS. **(A)** Funnel plot of the serum/plasma IGF-1 levels in patients with OSAS versus the control group. **(B)** Funnel plot of correlation coefficient between serum/plasma IGF-1 levels and AH. **(C)** Funnel plot of correlation coefficient between serum/plasma IGF-1 levels and minimum oxygen saturation.

Funnel plots were used to investigate the probability of publication bias in studies investigating the association between IGF-1 concentration and OSAHS, which appeared to be symmetrical. Begg’s (P=0.556) and Egger’s (P=0.307) tests did not reveal any publication bias in studies of patients with OSAHS (**Figure 5A**). A funnel plot was also drawn to evaluate the publication bias of the two meta-analyses with combined CORs (IGF-1 concentration versus AHI score and IGF-1 concentration versus minimum oxygen saturation). The funnel plots appeared to be symmetrical with no indication of publication bias (all P values >0.05, **Figures 5B, C**). Only three publications showed a correlation between IGF-1 concentration and ODI score; therefore, publication bias analysis was not conducted.

## 4 Discussion

IGF-1 levels are reportedly low in a variety of metabolic diseases and are believed to be involved in OSAHS progression (46). The serum/plasma IGF-1 concentrations of individuals from 34 studies were evaluated in this meta-analysis to compare the IGF-1 concentrations in patients with OSAHS and controls. According to these findings, adult patients with OSAHS had considerably lower plasma/serum IGF-1 levels than those in the control group. Similarly, serum IGF-1 concentrations in children with OSAHS were considerably reduced compared with those in the control group, similar to the results reported by Williams et al. and Nieminen et al. (37, 47). Bar et al. (48) reported that children with OSAHS had increased serum IGF-1 concentrations and body weights after adenotonsillectomy compared with children who did not undergo surgery, suggesting that OSAHS can alter serum IGF-1 levels in children. However, in our meta-analysis, no major difference in plasma IGF-1 concentrations was noted between children with and without OSAHS. The variance in the results might be due to the various sample sources and small sample sizes.

Notably, only one study by Gozal et al. (17) reported contradictory results in children, and found that IGF-1 levels were increased in children with OSAHS without evidence of cognitive dysfunction. The authors also pointed out that a significant increase in IGF-1 levels can occur among children who recovered their cognitive function, whereas in children without cognitive deficits, a decreased rather than increased IGF-1 concentration can be expected. This finding may be related to the inclusion of some particularity for the population resulting in a difference in their study. Cognitive function may affect plasma IGF-1 levels; however, the relationship between IGF-1 level and neurocognitive outcomes in children requires further investigation. Notably, serum IGF-1 concentrations in children with OSAHS were less reduced compared with those in affected adults, possibly owing to the synthesis and secretion of IGF-1 according to GH levels. GH levels are higher in young people than those in adults, and GH and IGF-1 are important modulators of energy metabolism in children (49). The difference in basal metabolism between children and adults may explain the difference in IGF-1 reduction between adults and children with OSAHS.

Furthermore, subgroup analyses based on different study designs revealed reduced plasma/serum IGF-1 concentrations in patients with OSAHS in cross-sectional, case-control, and cohort studies, indicating that in most reports, the serum/plasma IGF-1 concentrations in patients with OSAHS were reduced compared with those of control groups and that IGF-1 concentration may be related to OSAHS onset. Subsequently, a further meta-analysis of the CORs was performed between IGF-1 concentration and ODI score, AHI score, and minimum oxygen saturation. The results indicated that IGF-1 concentration had a moderate negative correlation with AHI score, a negative correlation with ODI score, and a moderate positive correlation with minimum oxygen saturation. AHI score, ODI score, and minimum oxygen saturation are all critical indicators for determining OSAHS severity, implying that IGF-1 levels are associated with OSAHS. Understanding the mechanism by which IGF-1 levels fluctuate in patients with OSAHS is critical because studies have shown that lower IGF-1 levels increase the risk of cardiovascular events following ischemic stroke and coronary intervention (50, 51). Tsai et al. (52) discovered that individuals with renal cell carcinoma with better prognoses had considerably greater baseline circulating IGF-1 concentrations than those in patients with worse prognoses. OSAHS is associated with high cardiovascular and cerebrovascular disease rates as well as malignancies (53–55). Serum/plasma IGF-1 monitoring may be important in determining the prognosis of patients with OSAHS. However, more clinical studies are needed to evaluate the correlations among cancer, OSAHS, and IGF-1 levels. Furthermore, this research has crucial clinical consequences, as the outcomes suggest that the metabolic levels of children and adults with OSAHS should be evaluated in addition to sleeping indicators assessed by PSG. Monitoring metabolism-related molecules in patients with OSAHS can further our understanding of IGF-1 variations among patients, thereby enhancing OSAHS risk assessment.

IGF-1 is a polypeptide protein that depends on GH effects to regulate insulin resistance (56). Fat and skeletal muscle are the primary target organs of the GH/IGF-1 axis, and play a key role in the pathophysiology of type 2 diabetes and obesity (57, 58). Patients with obesity and low IGF-1 concentrations have considerably more visceral obesity than patients with obesity and normal IGF-1 concentrations (59). OSAHS and obesity have a bidirectional relationship. Therefore, we conducted a subgroup analysis according to BMI and found that serum IGF-1 concentrations in patients with OSAHS and a mean BMI ≥ 30 and< 30 were lower than those of subjects in the control group, although the decreased serum IGF-1 levels in patients with OSAHS and a mean BMI≥30 were more prominent. This indicates that both obesity and OSAHS may lead to decreased IGF-1 levels *in vivo*, which aligns with the findings of previous research. Obesity mixed with OSAHS, meanwhile, creates a superposition effect, and subsequent drop in IGF-1 levels. However, we only found decreased plasma IGF-1 concentrations in patients with OSAHS who had a mean BMI≥30 in our analysis of plasma IGF-1 concentrations, possibly owing to the small number of studies on plasma IGF-1 concentration included in our analysis.

Moreover, from the serum IGF-1-related studies we discovered that serum IGF-1 concentrations in patients with OSAHS were reduced independent of disease severity or detection method, and that the serum IGF-1 level was more reduced in patients with OSAHS and high AHI scores, indicating that serum IGF-1 concentration is a possible risk factor for OSAHS. However, the results of the aforementioned plasma IGF-1 investigation found no variations in plasma IGF-1 concentrations between patients in the OSAHS and control groups among different AHI scores and sample testing methodologies. The discrepancy in the plasma/serum IGF-1 analysis results may be because there were fewer samples of plasma IGF-1 concentrations, and after subgroup analysis, each subgroup had fewer reports. The small sample sizes had a major impact on the outcomes. Further investigations on plasma IGF-1 concentrations and OSAHS are expected to be conducted in the future with larger sample sizes to confirm our findings. Surprisingly, decreased serum/plasma IGF-1 levels differed slightly according to ethnicity, implying that ethnicity may influence serum/plasma IGF-1 levels in patients with OSAHS. Polymorphisms in human *IGF-1* and *IGF-1* receptor genes have been identified. Genetic variations in the *IGF-1* gene control IGF-1 production (60). Grijalva-Avila et al. (61) hypothesized that the *IGF-1* RS6214 polymorphism is associated with higher serum IGF-1 expression in Latinos, and that the TT genotype is associated with obesity and body fat mass. Similarly, Chinese individuals with the *IGF-1* RS35767 AA genotype have reduced serum IGF-1 levels and a higher risk of type 2 diabetes (62).

IGF-1 and insulin have certain structural similarities, and the amino acid sequences of IGF-1 and insulin have a homology of approximately 50% (63). Numerous studies have revealed that IGF-1 enhances insulin sensitivity during glucose metabolism (64). The mechanism underlying the association between serum/plasma IGF-1 concentration and OSAHS remains unclear. Based on the sources, metabolism, and other influencing factors of IGF-1, several plausible biological explanations are summarized as follows. First, the majority of patients with OSAHS are complicated by insulin resistance. Respiratory disorders and intermittent hypoxia during nighttime sleep are the major causes of glucose metabolism disorders and insulin resistance, and their pathogenic effects are independent of confounding factors, such as obesity and age. Although OSAHS complicated by type 2 diabetes is clinically common, its detailed mechanism has not yet been elucidated. When patients are diagnosed with type 2 diabetes, the activity of their islet cells is often reduced by more than 50% relative to normal conditions, indicating that islet function impairment plays a key role in diabetes progression and occurs before diagnosis. IGF-1 and insulin have a synergistic effect, possibly indicating the level of insulin resistance in the human body. Clemmons et al. (65) revealed that a mouse model of insulin resistance could be created by knocking out the IGF-1 gene and injecting exogenous IGF-1 into mouse models, and found that the insulin resistance status of the models was considerably improved. Insulin resistance may be an indirect cause of the lower IGF-1 levels observed in patients with OSAHS. Second, repeated sleep apnea in patients with OSAHS can cause hypoxia, hypercapnia, acidosis, and even lower the affinity of insulin to its receptor. Insulin receptor activity (tyrosine kinase) also decreases simultaneously, leading to structural or functional defects in various enzymes in the glucose metabolic pathway (e.g., glucokinase) and impaired glucose metabolism in the body, which results in insulin resistance and IGF-1 deficiency. Meanwhile, long-term and repeated nocturnal hypoxic stress responses may increase the antagonistic products of insulin in the body, thereby aggravating insulin resistance (66, 67). Furthermore, even during the intermittent stages of stressful periods, the catecholamine and cortisol levels in patients with OSAHS are higher than those of individuals in the general population, thereby aggravating insulin resistance and lowering IGF-1 levels (68). Patients with OSAHS may experience repetitive arousal at night, resulting in sleep deprivation. Sleep deprivation can directly alter the transcription and post-transcriptional translation levels of IGF-1 mRNA *in vivo*, thereby lowering IGF-1 concentrations below the normal range and exacerbating insulin resistance. Reduced SWS time reportedly limits GH secretion, although IGF-1 secretion depends on the release of GH. GHs may play a major role in the stimulation of local IGF-1 synthesis (69). A study by Zhang and Du showed that recurrent hypoxemia affects GH and IGF-1 secretion, and in animals, hypoxia inhibits the release of GHs or biosynthesis (70). According to Han et al., hyperoxia also boosted the expression of IGF-1 and its type I receptor in rats (71). Consequently, we hypothesized that the GH/IGF-1 axis control problem caused a decrease in IGF-1 concentration produced by reduced SWS time and hypoxemia in patients with OSAHS. Our subsequent findings revealed that IGF-1 concentration was negatively correlated with AHI and ODI scores and positively correlated with minimum oxygen saturation, demonstrating that sleep hypoxia and sleep structure disorder have a significant impact on IGF-1 concentration.

The quality of the included studies, overall characteristics, experimental procedures, and other factors contributed to the heterogeneity of our meta-analysis. The overall heterogeneity in this study was substantial (I^2 =^ 94.2%); however, the sensitivity analysis highlighted that none of the publications had a considerable effect on the combined SMD, indicating that our findings are valid. According to the ethnicity and disease severity subgroup analysis, heterogeneity decreased although the I^2^ remained above 50% suggesting that unknown factors leading to heterogeneity, such as the use of different experimental kits and conditions or patient health status and lifestyle, may exist. Moreover, variations in sleep-disordered breathing examinations can cause heterogeneity, although most of the publications included in this study used standard PSG examinations; therefore, none of the subgroup analyses were based on the type of sleep-disordered breathing examinations. This meta-analysis presents certain advantages for detecting serum and plasma IGF-1 concentrations in patients with OSAHS. First, our findings imply that serum/plasma IGF-1 concentrations may be a possible biological biomarker for measuring OSAHS severity, which may assist clinicians in accurately diagnosing OSAHS and determining the disease severity. Second, this study is the first to combine the CORs for AHI score, ODI score, minimum oxygen saturation, and IGF-1 concentration and reveal an association between IGF-1 concentration and OSAHS from an evidence-based medicine perspective. Understanding the changes in serum/plasma IGF-1 concentrations in patients with OSAHS will aid researchers in furthering their understanding of the metabolic processes in OSAHS and in analyzing the OSAHS-related consequences. Galerneau et al. (72) concluded that IGF-1 concentration is a biomarker of OSAHS severity, and that using IGF-1 measurements in the personalized treatment of patients with OSAHS may be useful for grading disease severity and guiding specific interventions for OSAHS-related cardiometabolic risk. Third, this is the most thorough meta-analysis of the relevant literature conducted to date, resulting in reliable information. We included cross-sectional, case-control, and cohort studies to investigate variations in IGF-1 concentrations between patients with OSAHS and controls. Our study included a comparatively larger sample size (2,446 subjects) and more recent studies with medium-to high-quality publications. Finally, the serum and plasma IGF-1 concentrations were examined to eliminate sample origin-related confounding factors.

Despite the originality of our findings, this study has some limitations. First, this study may have included confounding variables, such as age, smoking history, lifestyle, alcohol consumption, sex, and diabetes. In particular, diabetes may be a major confounding factor for circulating IGF-1 levels. Moreover, OSA is more common in men, the older, and individuals with obesity compared with the general population (4). We were unable to control for the aforementioned confounding factors because the included articles did not provide data on past medical history, specific lifestyle factors, or analysis by sex, and no studies were conducted only in older patients with OSAHS. To a greater or lesser extent, several potential confounding factors might have influenced the final findings. Second, the electroencephalography fragmentation index and N3 sleep (SWS) were identified as important indicators in the evaluation of OSAHS; however, the included studies did not examine the direct relationship between serum/plasma IGF-1 levels, electroencephalography fragmentation index and N3 sleep (SWS). Third, we were unable to conclude a causal association between serum/plasma IGF-1 concentrations and OSAHS in this investigation because of the lack of an appropriate longitudinal cohort study. Fourth, most of the studies included were small sample studies comprised of< 100 cases, which was not sufficient to confirm an association between IGF-1 and OSAHS.

## 5 Conclusion

Patients with OSAHS had considerably lower plasma and serum IGF-1 concentrations when compared with those of the control group, which was negatively correlated with AHI and ODI scores and positively correlated with minimum oxygen saturation. The direct relationship between OSAHS and IGF-1 concentration was also affected by ethnicity and illness severity. Finally, further well-designed studies are required to evaluate the association between IGF-1 concentration and OSAHS risk.

## Data availability statement

The original contributions presented in the study are included in the article/Supplementary material. Further inquiries can be directed to the corresponding author.

## Author contributions

Data curation: JH and MY. Formal analysis: XL. Methodology: XL and MY. Project administration: XL. Resources: JH. Supervision: XL. Validation: JH. Writing-original draft: XL. All authors contributed to the article and approved the submitted version.

## Conflict of interest

The authors declare that the research was conducted in the absence of any commercial or financial relationships that could be construed as a potential conflict of interest.

## Publisher’s note

All claims expressed in this article are solely those of the authors and do not necessarily represent those of their affiliated organizations, or those of the publisher, the editors and the reviewers. Any product that may be evaluated in this article, or claim that may be made by its manufacturer, is not guaranteed or endorsed by the publisher.
